# Myofascial Trigger Points and Central Sensitization Signs, but No Anxiety, Are Shown in Women with Dysmenorrhea: A Case-Control Study

**DOI:** 10.3390/biology11111550

**Published:** 2022-10-23

**Authors:** Yennyt-Tatiana Hoyos-Calderon, Patricia Martínez-Merinero, Susana Nunez-Nagy, Daniel Pecos-Martín, César Calvo-Lobo, Carlos Romero-Morales, Vanesa Abuín-Porras, Ana Serrano-Imedio

**Affiliations:** 1 Hospital Universitario Infanta Sofía, 28703 Madrid, Spain; 2 Physiotherapy Department, Faculty of Medicine and Health Sciences, University of Alcalá, Alcalá de Henares, 28805 Madrid, Spain; 3 Faculty of Nursing, Physiotherapy and Podiatry, Universidad Complutense de Madrid, 28040 Madrid, Spain; 4 Faculty of Sport Sciences, Universidad Europea de Madrid, Villaviciosa de Odón, 28005 Madrid, Spain

**Keywords:** pelvic floor, dysmenorrhea, pain

## Abstract

**Simple Summary:**

In this study, a biologic tissue characteristic present in chronic pain conditions—myofascial trigger points—was assessed in a sample of women with primary dysmenorrhea (PD) and compared with a group of healthy subjects. The presence of alterations in cerebral pain management—central sensitization—and pain related anxiety, were also addressed. Women with PD showed symptoms of myofascial pain syndrome and central sensitization, but no pain-related anxiety. The results of the present study could guide physical therapy practitioners into more accurate techniques focused on identification and treatment of myofascial trigger points, taking into consideration the possible presence of central sensitization processes in these patients, which need to be identified and, if present, considered a therapeutic target.

**Abstract:**

Background primary dysmenorrhea (PD) is considered to be a cyclic chronic pelvic pain, with its onset in menstrual periods, often accompanied by the presence of myofascial trigger points (MTP). Most MTPs in subjects with chronic pelvic pain are in the inferior part of the abdomen, in the rectus abdominis (RA) area. Central sensitization is closely related to chronic pain processes. Previous studies in women with chronic pelvic pain reported central sensitization signs in their subjects, such as lower pain pressure threshold (PPT). Several authors agree that PPT in the tibialis anterior (TA) muscle, seems to be a reliable reference for signs of central sensitization. Amongst the factors that seem to accompany central sensitization, the presence of anxiety needs to be considered. The aim of the present study was to analyze the existence of hyperalgesic MTPs in RA, central sensitization signs and anxiety in women with PD, in comparison with a control group (CG). Methods: This study was designed following an observational, cross-sectional, case-control model. A total sample of 80 subjects was recruited trough social webs and advertising (PD n = 39) (CG n = 41). PPT in RA and AT was assessed bilaterally through algometry, and anxiety was evaluated through the State–Trait Anxiety Inventory. Results: Statistically significant differences (*p* < 0.001) were shown for NRS average and maximum increase, as well as lower bilaterally RA and TA PPT in favor of PD group compared to CG. State or trait STAI did not show any statistically significant differences (*p* > 0.05) between groups. Conclusions: In this study, women with PD reported symptoms of myofascial pain syndrome and central sensitization, when compared with healthy controls, without any sign of anxiety acting as a confounder for pain sensitivity.

## 1. Introduction

Dysmenorrhea is the most common gynecological cause of pain present in women of fertile age. It is considered to be a cyclic chronic pelvic pain, with its onset during menstrual periods. Pain is usually located in abdominal, pelvic, and superior pubic areas, occasionally reaching lumbar and lower limb areas [1]. Dysmenorrhea is classified as primary or secondary, depending on the presence of any previous pelvic pathology. Primary dysmenorrhea (PD) prevalence is estimated to be between 40% and 90% in women. Moreover, in severe cases, PD interferes with work and even school attendance [1]. Reported risk factors for PD are: diet, low socioeconomical level, sedentarism, age, low body mass index, smoking, alcohol intake, early menarche, long menstrual period and obesity, amongst others [2,3,4,5,6,7,8].

PD etiology is yet to be explored. The scientific literature points to high levels of prostaglandins present in woman with PD [9], triggering uterine contractions, muscle hypoxia and, consequently, pain. Prostaglandins have biological effects in most tissues, and are involved in muscle contraction and relaxation processes, as well as reproductive cycle events, such as ovulation, fertilization, and childbirth [9,10,11]. Dawood et al. [12], reported low levels of prostacyclin, a powerful inductor of uterine relaxation, in woman with PD. In addition, these authors observed a higher uterine basal tone in this population, with an elevated, asynchronous muscle contraction rhythm compared to a control group [12]. Treatment of PD is usually pharmacological. Non pharmacological treatment includes acupuncture, transcutaneous electrical nerve stimulation (TENS), relaxation techniques, exercise and physical therapy [13,14]. Nevertheless, 20–25% of women with PD do not respond to these treatments, suggesting that there might be a dysregulation of central nociceptive processing underlying this condition [15].

Chronic pelvic pain is often accompanied by myofascial pain syndrome symptoms [16,17]. This syndrome is defined by the presence of myofascial trigger points (MTP), which are described as hyperirritable nodules embedded in a tense muscle band [18]. The onset of MTPs can be related to direct muscle injuries, continuous overload or repetitive microtrauma. Pastore et al. [19] established the prevalence of myofascial symptoms in women with chronic pelvic pain at between 14% and 23%. Furthermore, the presence of MTPs can be also aggravated by anxiety, metabolic alterations, and other external factors [20,21,22,23]. Montenegro [24], carried out a study revealing that most MTPs in subjects with chronic pelvic pain are located in the inferior part of the abdomen, in the rectus abdominis (RA) area [25]. In addition, the main MTPs in this muscle can be located in the midway area of the line between the umbilicus and the pubic symphysis [26].

Central sensitization is closely related to chronic pain processes [27,28,29]. It is defined as a overstimulation of the central nervous system, originated by the constant nociceptive input from peripheric tissues [30]. Clinical manifestations may include local hyperalgesia and widespread pain [31]. Previous studies in women with other conditions causing chronic pelvic pain reported central sensitization signs in their subjects, such as lower pain pressure threshold (PPT). PPT is defined as the amount of pressure the subject describes as non-painful in a located point and is generally assessed by algometry [32]. Several authors agree that PPT in the tibialis anterior (TA) muscle seems to be a reliable reference for signs of central sensitization, considering it a site remote from the area of interest that could not be plausibly related, in terms of chronic pelvic pain extension, to the targeted area of the abdomen [33,34,35]. Amongst the factors that seem to accompany central sensitization, some psychological features need to be considered. Specifically, anxiety has been discussed by several authors as a possible confounder in PPT assessment [36]. Anxiety is associated with high alert levels involving perceived environmental threats and perceived pain [37]. Moreover, the presence of anxiety and other mental health factors, such as depression, concomitantly with dysmenorrhea has been analyzed extensively in recent scientific literature [38,39,40].

The aim of the present study was to study the presence of hyperalgesic MTPs in RA, central sensitization and related anxiety in women with PD, in comparison with a control group (CG).

## 2. Methods

### 2.1. Study Design

The study was designed with a case-control, cross-sectional model, following the STROBE declaration (Strengthening the Reporting of Observational Studies in Epidemiology) principles [41].

### 2.2. Participants

**Figure 1:**
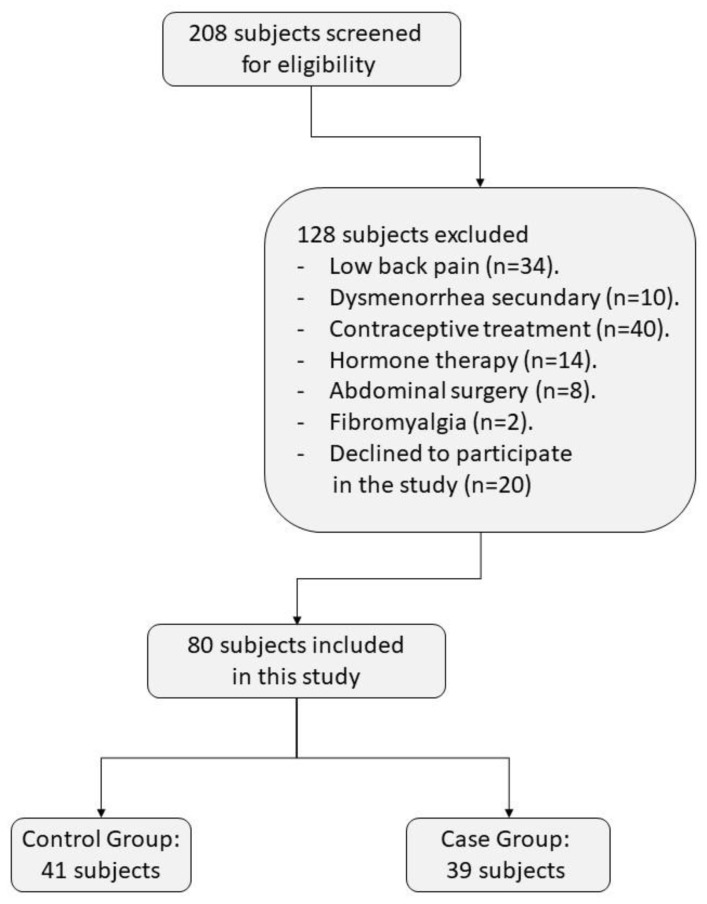
Flow chart.

Participants were recruited through social webs (Facebook, Instagram, Twitter) and advertisement though posters and flyers in several Spanish physiotherapy clinical sites related to the research team and/or the University of Alcalá de Henares. Advertising was also conducted at the Alcalá de Henares University campus. Inclusion criteria for the PD group were: (a) woman between 18 and 43 years; (b) diagnosed with PD by a gynecology specialist; and (c) nulliparous, whereas, for the CG, not reporting any severe menstrual pain episode during the past year was required. Exclusion criteria for both groups were: (a) pain medication intake 24h before evaluation that could interfere with the assessment; (b) secondary dysmenorrhea or any acute/chronic pelvic pathology that could generate pain in the lumbo-pelvic area in the past year; (c) RA injury; (d) pregnancy; and (e) contraceptive treatment/hormone therapy. A flow chart of the participants is shown in Figure 1.

### 2.3. Ethical Considerations

The study was authorized by the ethics committee of Alcala University, Madrid, Spain (CEIM/HU/2017/10) and was conducted between January 2018 and October 2019. The study respects the Declaration of Helsinki for human experimentation. All the participants in the study signed the informed consent form.

### 2.4. Procedure

All participants recruited through social webs and advertising were asked, via email, to complete a pre-screening form with demographic data, data of interest for the study (i.e., pregnancy history, chronic pelvic pain conditions, etc.), their menstrual pain history and whether they had a medical diagnosis of PD. Only participants with an official diagnosis of PD made by their gynecologist prior to the study were included. Additionally, homogeneity between groups was achieved through age quota matching of the PD group and the CG. Participants that met the inclusion criteria were asked to read and sign an informed consent form pre-assessment. All assessments were performed when women were not currently undergoing menstruation, to prevent menstrual cycle interferences. The evaluation took place in a private clinical center and was conducted by a certified physical therapist.

The presence of MTPs was determined through abdominal palpation in the RA, marking the most painful point. Then, algometry was applied to determine PPT in RA. An analog Fischer algometer (FPN100, URL myofib.com (accessed on 22 October 2022), Toledo, Spain) with a 5 kg rank, divided into 10 parts of ½ kg and a round rubber surface of 1 cm^2^, was employed in the assessment. Participants were asked to lie supine, with their arms along their body and a pillow below their knees to achieve maximum RA relaxation. Abdominal palpation was performed on both sides of the linea alba, following the muscle fibers from cranial to caudal and from medial to lateral. Furthermore, the examiner was placed on the homolateral side of the muscle that was being assessed, locating the muscle insertion in the rib cage and performing a longitudinal palpation towards the pubic insertion. The hyperalgesic spot was marked and then assessed with the algometer. The rubber surface was placed perpendicular to the muscle. Following that, the examiner applied pressure, increasing it gradually at 1 kg/s pace. Subjects were instructed to say “stop” when pressure started to become uncomfortable. Moreover, that same measure was repeated three times, and the mean of the trials was calculated and recorded. The same procedure was replicated for the TA assessment, with the examiner placed homolaterally to the muscle. The evaluation point was stablished 5cm below and 1cm lateral to the tibial tuberosity [35].

Typical menstrual pain was self-reported using a numeric rating scale. Participants were asked to grade their menstrual pain on a scale between 0 and 10, 0 being “absence of pain” and 10, “maximum pain intensity”.

Anxiety status was assessed through State–Trait Anxiety Inventory (STAI), which has 20 items for evaluating trait anxiety and 20 for state anxiety [42]. Every item has a 0 to 3 punctuation with a total maximum score of 60 indicating high levels of trait and state anxiety.

### 2.5. Statistical Analysis

SPSS 22.0 software (IBM SPSS Statistics for Windows; IBM Corp, New York, NY, USA.) was utilized for data analysis. An α error of 0.05 (95% confidence interval) and a desired power of 80% (β error of 0.2) were considered. Firstly, the Kolmogorov–Smirnov test was utilized to assess normality. Secondly, a descriptive analysis was performed for both groups, separately. Finally, a comparative analysis between both groups was carried out. For the parametric data, the mean ± standard deviation (SD) and Student’s *t*-test for independent samples were calculated. For the non-parametric data, the median ± interquartile range (IR) and Mann–Whitney *U* test were applied. In addition, Fisher´s exact test was used to analyze differences in medication use between both groups.

Sample size calculation was carried out using the difference between two independent groups through the G * Power 3.1.9.2 software based on the STAI-state score of a pilot study (n = 10) with 2 groups (mean ± SD), 5 women with dysmenorrhea (17.2 ± 8.16) and 5 control women (12.6 ± 8.20). An one-tailed hypothesis, an effect size of 0.56, an α error probability of 0.05, a power (1-β error probability) of 0.80 and an allocation ratio (N2/N1) of 1 were utilized for the sample size calculation. A total sample size of 80 subjects was estimated.

## 3. Results

Considering Table 1, sociodemographic data did not show statistically significant differences (*p* > 0.05) for age, weight, and BMI between groups. Nevertheless, statistically significant differences (*p* < 0.05) in height were shown, with higher numbers in women with dysmenorrhea compared to the control group.

Furthermore, there was a medication use difference (yes/no) between the PD group (35/4) and CG (17/24) (*p* < 0.001).

Regarding Table 2, statistically significant differences (*p* < 0.001) were shown for higher medication dosage, NRS average and maximum increase, as well as lower bilateral RA and TA PPT in favor of the PD group compared to CG. State or trait STAI did not show any statistically significant differences (*p* > 0.05) between groups.

## 4. Discussion

The purpose of this study was to analyze the presence of myofascial pain symptoms, central sensitization and anxiety in a sample of woman with PD compared to a group of women with no history of menstrual pain.

The results of the present study showed that woman with PD had a lower tolerance to pressure at RA hyperalgesic points. Several authors have addressed the presence of MTPs in chronic pelvic pain, but, to the authors’ best knowledge, this is the first study comparing changes in the myofascial system and nociceptive processing due to persistent pain in PD, compared to subjects with no PD. Huang & Liu [25] conducted a study with 65 patients diagnosed with PD, applying wet needling at 8 points along the RA and 4 on the lateral abdominal wall muscles. Assessment through algometry was not conducted in the Huang & Liu study. Moreover, the authors reported that tense muscle bands were not identified through palpation, but the presence of an MTP was assumed by the pain sensation in the area. Their results showed a statistically significant improvement in menstrual pain in their sample. Further studies with accurate tools are needed to determinate the exact target points for minimally invasive treatment, enhancing the efficacy of these interventions. Patil et al. [43] conducted a retrospective study, in 189 women with chronic pelvic pain, in which the authors reported the results of a myofascial intervention focused on the pelvic floor MTPs. The relation between pelvic floor muscles and abdominal wall muscles, acting as a synergic unit, is a common topic in scientific literature [44]. Therefore, the assessment and treatment of RA MPTs should be also considered in patients with chronic pelvic pain, such as patients with PD. Ross et al. [17], reported a case summary in which injections of local anesthetic in RA and pelvic-floor trigger points resulted in total resolution of the patient’s pain. These results have to be considered with caution, being a single case report.

Women in this study showed lower PPT values compared to the CG. Higher pressure pain sensitivity has been reported in chronic pelvic pain, as well as increased somatic pain sensitivity in deep tissues and viscera [45]. As-Sanie et al. [46] conducted an study, exploring central sensitization in woman with endometriosis compared to healthy controls, showing results coincident with those of the present study. Moreover, central sensitization has also been studied in other chronic pain conditions, such as fibromyalgia, knee osteoarthritis and low back pain [28,34,47,48]. Several authors have found a positive correlation between central sensitization and anxiety, which was not present in the present study. One possible reason for the discrepancy between results could be that fibromyalgia, low back pain and knee osteoarthritis [48,49,50] are considered as disabling conditions. Thus, the perception of disability could be an added reason for these higher levels of anxiety. The results of Wood et al. [45], showed a strong correlation between pain, disability an anxiety in their osteoarthritis patients. In comparison, PD, although painful and chronic, allows normal activity between menstrual cycles. Dos Santos et al. [47], in their study in patients suffering from painful temporomandibular disorder, also found no correlation between Central Sensitization Inventory scores and anxiety symptoms. Similar to PD, temporomandibular pain is not a condition considered as a cause of disability, thus, anxiety levels in these patients could, plausibly, not be raised by the presence of this pathology. In addition, both state STAI (mean PD = 12/CG = 12.9) and trait STAI (mean PD = 22/CG = 18.34) in the present study had values considered as “no or low anxiety” in each group. Therefore, the results for the lower PPT both in RA and TA are plausibly not influenced by an anxiety state.

### Limitations

This study has some limitations to be reported. Firstly, there was a significant difference in height between the case and the control group. To the authors’ best knowledge, there should not be any direct relation between height and any of the observed variables, but results should be interpreted cautiously in the light of this limitation. In addition, the assessment was limited to MTPs in the abdominal area, but other muscles also involved in chronic pelvic pain, such as pelvic floor muscles, were not evaluated. The aim of this study was to present a rapid non-invasive evaluation for certain MTPs and central sensitization. Further studies could address more complete evaluations in order to determine the best time and resources cost–benefit ratio for assessment. Finally, recruitment through social webs and advertising could be biased, as they cannot reach the whole possible target population.

The results of the present study could guide physical therapy practitioners into more accurate techniques focused on identification and treatment of MTPs, taking into account the possible presence of central sensitization processes in these patients, which need to be identified and, if present, considered a therapeutic target.

## 5. Conclusions

Myofascial pain syndrome symptoms, such as lower PPT in RA MPTs and central sensitization signs, are present in women with PD, when compared with a control group, without any sign of anxiety acting as a confounder for pain sensitivity.

## Figures and Tables

**Table 1:** Sociodemographic data for dysmenorrhea and control groups.

Data	Dysmenorrhea (n = 39)	Control (n = 41)	*p*-Value
Age, y	24.00 ± 6.00 ^†^	23.00 ± 9.50 ^†^	0.764 ^‡^
Weight, kg	59.79 ± 9.57 *	58.34 ± 11.50 *	0.467 **
Height, cm	166.76 ± 6.55 *	163.07 ± 6.21 *	0.011 **
BMI, kg/m^2^	21.49 ± 3.37 *	21.89 ± 2.78 *	0.558 **

Abbreviations: BMI, body mass index. * Mean ± standard deviation (SD) was applied. ** Student´s *t*-test for independent samples was performed. ^†^ Median ± interquartile range (IR) was used. ^‡^ Mann–Whitney *U* test was utilized.

**Table 2:** Descriptive data for dysmenorrhea and control groups.

Data	Dysmenorrhea (n = 39)	Control (n = 41)	*p*-Value
NRS, average	6.41 ± 1.35 *	3.75 ± 1.37 *	<0.001 **
NRS, maximum	8.35 ± 0.95 *	5.65 ± 1.68 *	<0.001 **
Left RA PPT, kg/m^2^	1.58 ± 0.40 *	2.40 ± 0.54 ^†^	<0.001 ^‡^
Right RA PPT, kg/m^2^	1.56 ± 0.36 *	2.38 ± 0.62 *	<0.001 **
Left TA PPT, kg/m^2^	2.82 ± 0.79 *	4.50 ± 1.07 ^†^	<0.001 ^‡^
Right TA PPT, kg/m2	3.23 ± 1.23 ^†^	4.49 ± 0.72 *	<0.001 ^‡^
STAI, anxiety-state	12.00 ± 14.00 ^†^	12.90 ± 9.10 *	0.352 ^‡^
STAI, anxiety-trait	22.00 ± 9.00 ^†^	18.34 ± 9.13 *	0.053 ^‡^

Abbreviations: RA, rectus anterior; STAI, state–trait anxiety inventory; TA, tibialis anterior; NRS, numeric rating scale. * Mean ± standard deviation (SD) was applied. ** Student´s *t*-test for independent samples was performed. ^†^ Median ± interquartile range (IR) was used. ^‡^ Mann–Whitney *U* test was utilized.

## Data Availability

Data available under reasonable request.
